# High genetic diversity and connectivity in *Colossoma macropomum* in the Amazon basin revealed by microsatellite markers

**DOI:** 10.1590/1678-4685-GMB-2015-0222

**Published:** 2017-02-06

**Authors:** Paola Fazzi-Gomes, Sávio Guerreiro, Glauber David Almeida Palheta, Nuno Filipe Alves Correa de Melo, Sidney Santos, Igor Hamoy

**Affiliations:** 1 Programa de Pós Graduação em Aquicultura e Recursos Aquáticos Tropicais, Instituto Socioambiental e dos Recursos Hídricos (ISARH), Universidade Federal Rural da Amazônia (UFRA), Belém, PA, Brazil; 2 Instituto Socioambiental e dos Recursos Hídricos (ISARH), Universidade Federal Rural da Amazônia (UFRA), Belém, PA, Brazil; 3 Laboratório de Genética Humana e Médica, Instituto de Ciências Biológicas, Universidade Federal do Pará (UFPA), Belém, PA, Brazil; 4 Universidade Federal Rural da Amazônia (UFRA), Campus Capanema, Capanema, PA, Brazil

**Keywords:** Tambaqui, genetic variability, gene flow, genetic structure, single sequence repeats

## Abstract

*Colossoma macropomum* is the second largest scaled fish of the Amazon. It is economically important for commercial fisheries and for aquaculture, but few studies have examined the diversity and genetic structure of natural populations of this species. The aim of this study was to investigate the levels of genetic variability and connectivity that exist between three natural populations of *C. macropomum* from the Amazon basin. In total, 247 samples were collected from the municipalities of Tefé, Manaus, and Santarém. The populations were genotyped using a panel of 12 multiplex microsatellite markers. The genetic diversity found in these populations was high and similar to other populations described in the literature. These populations showed a pattern of high gene flow associated with the lack of a genetic structure pattern, indicating that the number of migrants per generation and recent migration rates are high. The values of the F_ST_, R_ST_, and exact test of differentiation were not significant for pairwise comparisons between populations. The Bayesian population clustering analysis indicated a single population. Thus, the data provide evidence for high genetic diversity and high gene flow among *C. macropomum* populations in the investigated region of the Amazon basin. This information is important for programs aiming at the conservation of natural populations.

*Colossoma macropomum* (Cuvier, 1818), commonly known as tambaqui in Brazil and as gamitana in Peru, is the largest characid fish of the Amazon basin, belonging to the family Characidae, subfamily Serrasalminae. *C. macropomum* is a tropical fish species found in the Orinoco and Amazon River basins as well as in their major tributaries. It reaches an average length of 1 m and weight of 30 kg (Araújo-Lima and Goulding, 1998). Tambaqui is a frugivorous fish and a key seed disperser for many plant species in the Amazon floodplain (Anderson *et al*., 2011).

*C. macropomum*, one of the most widely sold fish in regional markets in the Amazon, has been exploited commercially since the late 19^th^ century (Santos *et al*., 2007). Additionally, it is the most cultivated Neotropical fish in Brazil. There are strong indications, including reduction in supplies at Amazonian markets and continual reduction in the size of fish caught, that the natural populations of tambaqui are suffering from overexploitation (Araújo-Lima and Ruffino, 2004).

Due to its economic importance in the Amazon, it is essential to understand the levels of genetic variability and genetic structure patterns present in natural populations to develop management strategies that can keep in check the loss of genetic diversity among natural populations (Aguiar *et al*., 2013).

Microsatellite DNA is one of the best molecular markers for estimating the genetic diversity of natural populations and the genetic differentiation between closely related populations (Putnam and Carbone, 2014). The only previous study using microsatellite markers to evaluate the genetic variability and population structure of natural populations of *C. macropomum* in the Amazon basin is that of Aldea-Guevara *et al*. (2013). Hence, the aim of this investigation was to determine the level of genetic variability and population differentiation among samples of *C. macropomum* along the Amazon River using microsatellite markers.

A total of 247 samples of *C. macropomum* were caught with the support of artisanal fishermen in the municipalities of Tefé (95), Manaus (89) and Santarém (63) (Figure 1) in the Amazon Basin, Brazil. A sample of 2 g of muscle tissue was collected from each individual, preserved in 95% ethanol, and stored at 4 °C. Total genomic DNA was extracted from tissue digested in a proteinase K/sodium dodecyl sulfate solution at 54 °C for 4 h. DNA was purified using the standard phenol/chloroform method (Sambrook and Russell, 2001) and quantified using a NanoDrop™ ND-1000 spectrophotometer (Thermo Scientific).

**Figure 1:**
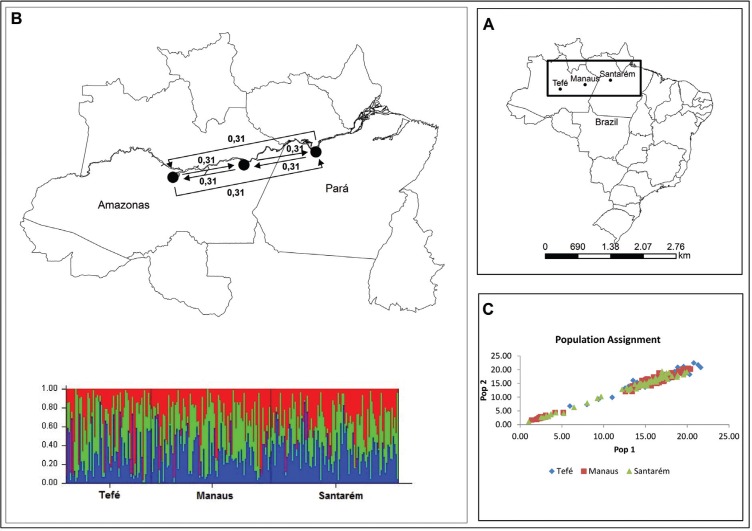
Populations of *Colossoma macropomum* in the Amazon studied. (A) Map illustrating the localization of *Colossoma macropomum* populations in the Amazon basin. (B) Nonsignificant asymmetry of migration rates at k = 3, arrows indicate direction and rate of recent migration rates; estimates were obtained with BIMr and Bar plots from Structure (below) without clustering of the data (C) Population assignment test done in GenAlEx to determine the likelihood of inclusion of the individuals in each population.

The genotyping protocol was described in Hamoy and Santos (2012), including the multiplex panel of 12 microsatellite markers for *C. macropomum* (Table 1). To identify possible genotyping errors, such as stuttering bands, which are common in dinucleotide microsatellites, the program Micro-Checker (van Oosterhout *et al*., 2004) was used.

**Table 1:** Statistics for individual loci and combined genotype among *Colossoma macropomum* populations in the Amazon basin.

*Loci*	Tefé					Manaus					Santarém				
	(N=95)					(N=89)					(N=63)				
	H_O_	H_E_	N_A_	A_R_	PIC	H_O_	H_E_	N_A_	A_R_	PIC	H_O_	H_E_	N_A_	A_R_	PIC
Cmacrμ01	0.64	0.75	7	6.3	0.70	0.63	0.72	6	5.7	0.67	0.57	0.71	8	7.3	0.67
Cmacrμ02*	0.59	0.79	10	8.9	0.76	0.56	0.85	12	10.6	0.83	0.43	0.83	7	7.0	0.68
Cmacrμ03	0.75	0.78	11	9.4	0.74	0.67	0.78	10	9.4	0.75	0.69	0.83	12	11.5	0.75
Cmacrμ04	0.79	0.82	8	7.9	0.79	0.82	0.81	11	9.7	0.78	0.88	0.83	7	6.9	0.72
Cmacrμ05	0.83	0.79	8	7.5	0.75	0.78	0.80	7	6.9	0.76	0.82	0.80	9	8.5	0.77
Cmacrμ06	0.73	0.73	7	6.3	0.68	0.68	0.76	7	6.2	0.72	0.77	0.78	6	5.7	0.67
Cmacrμ07	0.76	0.88	17	15.8	0.86	0.85	0.88	15	13.9	0.87	0.84	0.86	14	13.4	0.82
Cmacrμ08	0.75	0.79	9	8.8	0.75	0.69	0.79	10	9.2	0.74	0.68	0.81	8	7.9	0.69
Cmacrμ09	0.88	0.80	9	8.7	0.77	0.75	0.77	9	8.5	0.73	0.85	0.76	9	8.9	0.78
Cmacrμ10	0.81	0.77	6	5.9	0.73	0.71	0.76	6	5.9	0.71	0.75	0.80	6	5.9	0.63
Cmacrμ12	0.75	0.87	10	9.9	0.85	0.77	0.88	11	10.5	0.86	0.75	0.86	11	10.7	0.83
Cmacrμ13	0.81	0.78	11	9.2	0.75	0.80	0.80	8	7.9	0.77	0.88	0.81	9	8.5	0.83
Average	0.75	0.79	9.4	8.7	0.76	0.72	0.80	9.3	8.7	0.76	0.74	0.80	8.8	8.5	0.73

N - number of individuals, H_O_ - observed heterozygosity, H_E_ - expected heterozygosity, N_A_ - number of alleles per locus, A_R_ - allelic richness, PIC - polymorphism information content,

* - statistical significance after Bonferroni correction for Hardy–Weinberg equilibrium.

Allele frequencies of each marker in the different populations were calculated using Fstat 2.9.3.2 (Goudet, 2001). The observed (H_O_) and expected (H_E_) heterozygosity and possible deviations from Hardy-Weinberg equilibrium (HWE) were calculated with the program Arlequin 3.5.1.3 (Excoffier and Lischer, 2010), followed by Bonferroni correction (Rice, 1989) of the p-values found (adjusted p-value < 0.0041). Other parameters of genetic variability, such as the number of alleles per locus (N_A_) and allelic richness (A_R_) (El Mousadik and Petit, 1996), were estimated using Fstat 2.9.3.2 (Goudet, 2001). Polymorphism information content (PIC) was estimated using the program Cervus 3.0 (Kalinowski *et al*., 2007).

To investigate how genetic variability is distributed across different populations, populations from Tefé and Manaus were randomly grouped, with the population from Santarém forming another group, and analysis of molecular variance (AMOVA) (Excoffier *et al*., 1992) was performed using Arlequin 3.5.1.3 (Excoffier and Lischer, 2010).

Inter-population genetic differentiation was assessed using the R_ST_ (Slatkin, 1995) and F_ST_ (Weir and Cockerham, 1984) parameters, as well as the exact test of population differentiation (Raymond and Rousset, 1995), which has not previously been used to compare populations. These analyses were performed using Arlequin 3.5.1.3 software (Excoffier and Lischer, 2010).

Structure 2.2 software (Pritchard *et al*., 2000) was used in this study. This program uses Bayesian analysis to infer the number of genetically homogeneous populations (K) most likely (mean of Ln prob) to occur in the database analyzed. The actual value for K (that most likely explains the population database) is obtained after performing a Markov Chain Monte Carlo (MCMC) analysis with multiple simulations between the clusters assigned by the program and the variation in K entered in the program; our analysis used 100,000 simulations. The number of structured populations (K) was estimated based on 10 replications for each K (from 1 to 3). The logarithm of the probability of the data (lnP(D K); Pritchard *et al*., 2000) and estimates of Δk (Evanno *et al*., 2005) were evaluated using Structure Harvester (Earl and Von Holdt, 2012). The program Clumpp v.1.1.2 (Jakobsson and Rosenberg, 2007) was used to align the 10 repetitions of the best K.

The gene flow between populations was inferred by calculating the Nm (number of migrants per generation) using the private alleles method (Slatkin, 1985), which was implemented using the Genepop 4.0.10 program (Rousset, 2008). The number of private alleles (only present in one population) that show a linear correlation with Nm was determined for all markers in the four populations investigated here, and the average frequency of private alleles between paired populations was estimated. The population assignment from the program GenAlEx 6.5 (Peakall and Smouse, 2012) was used to determine the likelihood of inclusion of the individuals in each population.

The software BIMr 1.0 was used to detect the recent migration rates (m) and the possibility of asymmetrical rates based on the Bayesian assignment test (Faubet and Gaggiotti, 2008); this allows for departures from HWE within populations and uses the F-model and MCMC analysis. The F-model improves estimation of allele frequencies when genetic differentiation is weak, which allows BIMr to estimate rates of migration between populations that are weakly differentiated. We ran 20 replicates, with a total of 100,000 iterations each, and then collected 20,000 samples. For each replicate, we first performed MCMC analysis for 20 short pilot runs of 1000 iterations each.

The Micro-Checker program did not detect evidence of genotyping errors at any of the loci. Only the Cmacrμ02 locus differed significantly from the HWE after Bonferroni correction in the three populations analyzed, all of which showed an excess of homozygotes, suggesting the presence of null alleles in this marker. In total, 145 alleles were detected among the 12 loci analyzed, with H_O_ values ranging from 0.43 (Cmacrμ02) to 0.88 (Cmacrμ09, Cmacrμ04 and Cmacrμ13), N_A_ values ranging from 6 (Cmacrμ01, Cmacrμ06, Cmacrμ10) to 17 (Cmacrμ07) alleles, A_R_ values ranging from 5.7 (Cmacrμ01) to 15.9 (Cmacrμ07), and PIC values ranging from 0.63 Cmacrμ10 to 0.87 (Cmacrμ07) (Table 1). Based on these results, Cmacrμ07 was the most informative marker in this study. AMOVA revealed that 92% of all genetic variation found in the hierarchy was between individuals, independent of the populations or groups, 7% was between individuals within populations, 0.8% between populations within groups, and 0.2% between groups, showing that most of the genetic variation does not form distinct groups.

The R_ST_ and F_ST_ values between paired populations were equal to zero and not significant (P-value > 0.05). The results of the exact test of population differentiation were also not significant at a level of 5% when we compared the populations, this indicating the absence of genetic differentiation between pairs of populations. The results of the Structure program showed that the highest average likelihood was a single cluster K=1 (Figure 1) in these situations, so there was no need to perform the Evanno test.

The Nm values between populations were high for Tefé/Santarém (11.7), Tefé/Manaus (11.1) and Manaus/Santarém (8.3). The studied populations had few private alleles, with four in Tefé, two in Manaus and five in Santarém, among the total of 145 alleles found. The average frequency of private alleles between populations was also small, with values close to 0.1. The m values between populations were high and very similar, with all values close to 31%. In addition, the Bayesian approach implemented in BIMr did not identify significant asymmetric gene flow between populations (Figure 1). The population assignment test showed that only 35% of samples are outcomes of self-population, whereas 66% are outcomes of other populations (Figure 1).

For an intensively exploited species such as the tambaqui, the discovery of the level of genetic diversity within natural populations is critical to the prognosis of the viability of the species. The values for genetic diversity found in the populations analyzed with these microsatellite markers were high (H_O mean_ > 0.70) and similar to data reported in the literature for these same markers used for different populations in the Amazon region (Hamoy *et al*., 2011, Hamoy and Santos, 2012; Aldea-Guevara *et al*., 2013). Other studies have evaluated the levels of genetic variability in natural populations of tambaqui in the Amazon basin using different microsatellite and mtDNA markers and have also observed high levels of genetic variability (Santos *et al*., 2007, 2009; Molecular Ecology Resources Primer Development Consortium *et al*., 2013). Genetic studies of other Amazonian fish species that have been intensely exploited, such as *Arapaima gigas*, have shown a similar pattern of high genetic variability, including studies reported by Hrbek *et al*. (2005, 2007) and Hamoy *et al*. (2008), that used both microsatellite and mtDNA markers.

The present results indicate that the populations studied are genetically homogeneous. Similarly, Aldea-Guevara *et al*. (2013) employed the same microsatellite markers used in this study, and a Bayesian approach did not detect population differentiation in four lakes from the Amazon basin in Peru. However, the Bayesian approach using thermodynamic integration revealed non-panmictic populations with a stepping-stone migration pattern among those lakes.

The results showing high gene flow are similar to those found by Santos *et al*. (2007) using a control region of mtDNA in natural populations of *C. macropomum* from the Amazon basin. That study also found little genetic differentiation and high gene flow between populations, leading the authors to propose that these populations form a large panmictic population in the tributary system of the Amazon River. Using mtDNA control regions, Farias *et al*. (2010) showed that the tributaries of the Madeira River, which separate Bolivia's Amazon basin, do not represent an effective barrier against gene flow of *C. macropomum* populations from these basins, although genetic differences were found to exist between them

These results seem consistent with the biology of *C. macropomum*, which is a species that exhibits migratory behavior in search of food, protection, and reproduction. *C. macropomum*, which is a frugivorous species, is an important long-distance seed disperser for various species of plants in the Amazon floodplain, and overexploitation of *C. macropomum* can be considered a threat to the diversity of these plants (Anderson *et al*., 2009, 2011).

Our results suggest the absence of a genetic structure in *C. macropomum*, with high genetic variability and high gene flow in the Amazon basin. However, this current scenario does not guarantee the maintenance of this diversity over time. Only the maintenance and improvement of public policies regulating capture and management can ensure the viability of this important species. Another needed approach includes an examination of the levels of genetic variability in unstudied populations of *C. macropomum* to determine whether the maintenance of this pattern of genetic structure is coupled to high genetic diversity and high gene flow.
